# Anaphylactic reaction to carboplatin diagnosed by skin testing—a reliable tool in platinum-based immediate-type hypersensitivity reactions

**DOI:** 10.1007/s10354-022-00938-x

**Published:** 2022-05-20

**Authors:** Elias Marquart, Ahmad Jalili, Nadine Mothes-Luksch, Stephan N. Wagner, Tamar Kinaciyan

**Affiliations:** 1 Department of Dermatology, Medical University of Vienna, Waehringer Guertel 18–20, 1090 Vienna, Austria; 2 Dermatology & Skin Care, Bürgenstock Medical Center, Obbürgen, Switzerland

**Keywords:** Drug-related hypersensitivity reactions, Immediate-type allergic reaction, Platins, Allergologic work-up, Melanoma, Arzneimittel-Überempfindlichkeitsreaktionen, Allergische Reaktion vom Soforttyp, Platine, Allergologische Abklärung, Melanom

## Abstract

Immediate-type hypersensitivity reactions (IHRs) to carboplatin (CA) are most commonly reported in ovarian cancer patients. A 54-year-old woman with stage IV melanoma suffering from metastasis in the entire right lower extremity was presented to our allergy outpatient clinic for diagnostic work-up due to an anaphylactic reaction with palmoplantar erythema, conjunctivitis along with facial erythema, and an incipient decrease in blood pressure during a chemotherapy regimen with dacarbazine and carboplatin upon re-administration. A subsequently carried out allergological work-up with skin testing (ST) revealed CA to be the culprit drug, whereas cisplatin (CI) was confirmed to be a safe alternative for the patient for following treatments. Here, we report a case of an IHR to carboplatin in a melanoma patient, with CI serving as a safe alternative diagnosed by skin testing.

## Abstract (de)

Allergische Reaktionen vom Soforttyp auf Carboplatin werden meist bei Patientinnen mit Ovarialkarzinom beschrieben. Eine 54-jährige Patientin mit einem Melanom im Stadium IV und einer Metastasierung im Bereich der gesamten rechten unteren Extremität wurde in unserer Allergieambulanz zur diagnostischen Abklärung vorstellig. Anamnestisch hatte sie während einer Chemotherapie mit Dacarbazin und Carboplatin nach neuerlicher Verabreichung eine anaphylaktische Reaktion mit einem palmoplantaren und fazialen Erythem, einer Konjunktivitis sowie beginnendem Blutdruckabfall entwickelt. Die allergologische Abklärung mit Hauttestungen konnte Carboplatin als Auslöser identifizieren. Wir berichten hier über einen Fall einer allergischen Soforttyp-Reaktion auf Carboplatin bei einer Melanompatientin, die mittels Hauttestungen bestätigt werden konnte, während sich Cisplatin hierbei als sichere Alternative erwies.

## Introduction

Platin-based chemotherapeutic agents—platin salts (PS) such as carboplatin (CA), cisplatin (CI), and oxaliplatin (OX)—are mainly used for the treatment of gynecological neoplasms, bronchial and squamous cell carcinoma, and melanoma. Immediate-type hypersensitivity reactions (IHRs) and anaphylaxis to CA are most commonly reported in ovarian cancer patients [1, 2]. Here, to the best of our knowledge, we report the first case of an IHR to CA in a stage IV melanoma patient that was diagnosed by skin testing (ST).

This case report predates the establishment of immune checkpoint inhibitors as standard therapy for the treatment of melanoma. Nevertheless, we believe that awareness of allergic reactions to PS is important even in these patients, as platins may continue to be used for melanoma therapy in the event of treatment failure or wherever checkpoint inhibitors are not available.

## Case report

A 54-year-old woman presented to our allergy outpatient clinic with stage IV melanoma and disseminated metastasis in the entire right thigh with ulcerations. Initial treatment consisted of isolated limb perfusion with melphalan, low- and high-dose interferon alpha, as well as radiotherapy without efficacy. Subsequently, a modified treatment regimen with cisplatin, vinblastine, dacarbazine (CVD) according to Legha et al. [1] with temozolomide, vindesine, and carboplatin was initiated. Complete resolution of all ulcerations and the entire metastatic swelling of the right thigh was reached after nine cycles; therefore, chemotherapy was suspended.

Seven months later, a CT scan at a follow-up visit revealed cerebral metastasis. Therefore, after Gamma Knife treatment (Leksell Gamma Knife® Perfexion™ | Elekta Sweden used at the Department of Neurosurgery Medical University of Vienna, Vienna Austria), chemotherapy was reinitiated, but temozolomide was replaced with dacarbazine. The patient received ondansetron, dexamethasone, mannitol 20%, dacarbazine, and carboplatin. During the administration, she developed palmoplantar erythema with tingling, conjunctivitis along with facial erythema, and an incipient decrease in blood pressure. Chemotherapy was immediately discontinued. Symptoms resolved soon after treatment with intravenous corticosteroids and antihistamines. Because of previous reports about immediate-type hypersensitivity reactions (IHR) for all administered drugs (mannitol, dacarbazine, carboplatin), an allergological work-up was carried out for all three substances, and in a second step for CI as an alternative platin (Table 1).

**Table 1:** Overview of allergological work-up

Medication	Dacarbazine	Carboplatin	Cisplatin	Mannitol 20%	Temozolomide oral preparation
Prick test	(10 mg/ml) negative	(10 mg/ml) negative	(1 mg/ml) negative	(as is) negative	NA
Scratch test	NA	NA	NA	NA	Negative
IDT 0.01 mg/ml	Negative	Positive	Negative	ND	NA
IDT 0.1 mg/ml	Negative	Positive	Negative	False positive	NA

**Fig. 1:**
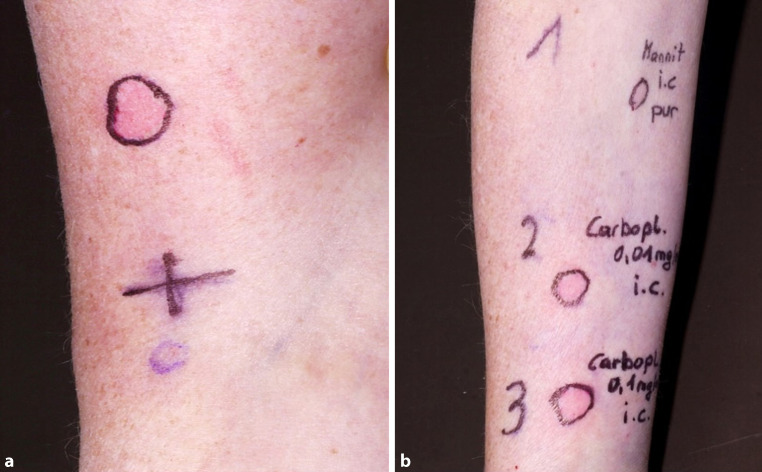
**a** Positive results of prick testing and intradermal testing (IDT) for histamine controls (10 mm wheal), **b** IDT testing of undiluted mannitol 20%—was an irritant test reaction as the control person reacted similarly—and positive IDT to carboplatin in two different dilutions—0.01 mg/ml (9 mm wheal), 0.1 mg/ml (11 mm wheal). Negative skin test results are not shown

Prick testing was performed using the therapeutic concentrations of dacarbazine (DACARBAZINE®, Medac GmBH), carboplatin (CARBOPLATIN®, EBEWE Pharma), and cisplatin (CISPLATIN®, EBEWE Pharma). For intradermal testing (IDT), the therapeutic concentration of dacarbazine, carboplatin and cisplatin was diluted as 1:1000; 1:100. IDT with undiluted mannitol (Mannitol Viaflo 20%®, Baxter Healthcare GmbH)—not shown in the table but in Fig. 1a,b was an irritant test reaction as the control person reacted similarly

*ND* not determined, *NA* not applicable

Scratch testing with temozolomide oral preparation and prick testing (PT) with other involved drugs resulted negative for all tested substances. Intradermal testing (IDT; Fig. 1a,b) performed on separate visits resulted in an immediate positive reaction for CA (wheal and flare reaction after 20 min) but not for CI (data not shown). Although specific IgE antibodies to CA were not checked, we are strongly convinced that our patient suffered from an IgE-mediated immediate-type hypersensitivity reaction to CA, based on the appearance of the reaction at the second treatment cycle, the clinical course with rapid development, increasing severity upon re-exposition, and positive IDT. As a result, we substituted CA with CI and were able to continue the treatment of our patient without any further adverse effects.

## Discussion

In particular, the occurrence of IHR to CA is associated with high dosages (> 650 mg), high numbers of treatments  (> six), and long treatment-free intervals (> 13 months) between each chemotherapy cycle [2]. Common symptoms include mild allergic reactions (erythema, itching, facial flushing), but more severe reactions such as hypotension, dyspnea, or chest pain have also been reported [3].

Current guidelines of the ENDA/EAACI Drug Allergy Interest Group by Brockow et al. [4] suggest non-irritant IDT concentrations for various drugs including CI and CA, but not dacarbazine or mannitol. As our allergological work-up predates these guidelines, we used an in-house test protocol with nonirritant test concentrations starting with higher dilutions than suggested, as shown in Table 1. Still, our CA and CI test concentrations for PT and IDT are in accordance with current guidelines, with the exception of the IDT concentrations for CA, which even used a 10- and 100-times higher diluted test concentration. To exclude false-positive irritant test reactions for drugs where no test guidelines are available, control persons should be tested as a rule. As IDT with undiluted mannitol 20% was positive in our patient, we tested a nonallergic, healthy control person who developed a similar skin reaction. Therefore, we concluded this was an irritant reaction.

After careful reevaluation and given the advanced stage of melanoma in our patient, the treatment of choice was substitution of CA with CI according to our ST results and previous reports [5]. Since an equivalent alternative was available in CI, we refrained from desensitization procedures prior to each administration of CA [6–8].

## Conclusion

In conclusion, the present case highlights i) the increasing severity of an immediate-type CA hypersensitivity reaction with further treatment in a stage IV melanoma patient, ii) demonstrates that skin testing is a very reliable tool to clarify CA allergy and exclude cross-reactivity to other PS [9], and iii) CI serves as an adequate substitute for CA [10, 11].
